# Ethylene-mediated signaling confers thermotolerance and regulates transcript levels of heat shock factors in rice seedlings under heat stress

**DOI:** 10.1186/s40529-019-0272-z

**Published:** 2019-09-23

**Authors:** Yu-Sian Wu, Chin-Ying Yang

**Affiliations:** Department of Agronomy, National Chung Hsing University, Taichung, 40227 Taiwan

**Keywords:** Heat stress, Ethylene, Heat shock factor, Thermotolerance, Antioxidant enzyme

## Abstract

**Background:**

Agriculture is highly dependent on climate. Increases in temperature caused by global warming pose challenges for crop production. Heat stress induces oxidative damage to cell membranes and then causes cell death. Plants have developed various responses to elevated temperatures, including hormone signaling pathways and heat shock factors that elevate their thermotolerance. In response to heat stress, the gaseous hormone ethylene is produced through regulation of the expression of signaling-related genes to modulate resource allocation dynamics. For comprehensive understanding of the role of ethylene, this study used an ethylene precursor to analyze the ethylene signaling pathway involved in adjustment of the homeostasis of the antioxidant system and to evaluate heat shock factor expression in rice seedlings under heat stress.

**Results:**

Levels of cell membrane oxidation and ion leakage were reduced in rice seedlings under heat treatment combined with ethylene precursor treatment, conferring enhanced thermotolerance. Reduction of the fresh weight and chlorophyll a/b ratio in rice seedlings was lower in rice seedlings under heat stress with ethylene precursor treatment than in those under heat stress only. Moreover, reduction of antioxidant response caused by heat stress was ameliorated by treatment with ethylene precursors such as catalase and total peroxidase. Quantitative reverse transcriptase-polymerase chain reaction showed higher expression levels of heat shock factors such as *HSFA1a* and *HSFA2a*, *c*, *d*, *e*, and *f* and ethylene-signaling-related genes such as *ethylene insensitive 2*, *ethylene insensitive*-*like 1*, and *ethylene insensitive*-*like* 2 in rice seedlings under heat stress with ethylene precursor treatment than in rice seedlings under heat stress only.

**Conclusion:**

Ethylene-mediated signaling was involved in the reduction of oxidative damage, maintenance of chlorophyll content, and enhancement of thermotolerance in rice seedlings under heat stress. Furthermore, this study revealed heat shock factors and ethylene-signaling-related genes involved in complex network regulation that confers thermotolerance to rice seedlings.

## Background

Due to climate change and rising global temperatures, crops are frequently exposed to heat stress, which subsequently threatens food security. Heat stress affects crop growth, photosynthesis, and membrane stability and reduces financial yield (Kotak et al. 2007; Schauberger et al. 2017; Wahid et al. 2007). To adapt to high-temperature conditions, plants have evolved two mechanisms to face environmental pressure. One mechanism is intrinsic thermotolerance; plants have the inherent ability to survive under temperatures higher than the optimal temperature for growth. The other mechanism is acquired thermotolerance; plants have the ability to grow under lethally high temperatures following acclimatization to such temperatures over a short period (Liu and Charng 2012; Song et al. 2012). Studies have indicated that survival rates are enhanced by exposing rice, maize, barley, and *Arabidopsis* plants to extreme temperatures for short periods and allowing subsequent recovery (Charng et al. 2007; Hong and Vierling 2000; Maestri et al. 2002; Queitsch et al. 2000).

The plant hormone ethylene is reportedly a key regulator involved in abiotic or biotic stress signaling. Interaction between ethylene and a receptor complex triggers inactivation of constitutive triple response 1 kinase, which leads initially to dephosphorylation of ethylene insensitive 2 (Savada et al. 2017) by an as-yet-unidentified phosphatase and subsequently to cleavage of the C-terminal of EIN2 and its translocation to the nucleus, where it regulates EIN3/EIL1 activation. EIN3/EIL1 proteins further regulate ethylene response factors (ERFs) (Yoshida et al. 2011) and promote the transcriptional cascade that activates and represses hundreds of genes under the ethylene signaling pathway (Müller and Munné-Bosch 2015). The survival rates of *ethylene receptor 1* and *EIN2* mutants were lower than those of wild-type *Arabidopsis* under heat stress; this finding supported that ethylene signaling is involved in the basal thermotolerance mechanism (Larkindale and Knight 2002; Larkindale et al. 2005).

Heat stress transcription factors (HSFs)—which comprise the plant intracellular response to heat stress—bind to the promoter element of heat shock proteins, which function as molecular chaperones that prevent aggregation of proteins and facilitate refolding of heat-damaged proteins. The gene diversity of HSF families has been identified as 21 in *Arabidopsis*, 25 in rice (*Oryza sativa*), 30 in maize (*Zea mays*), and 24 in millet (Guo et al. 2008, 2016; Scharf et al. 2012). According to HSF protein structure characterization and phylogenetic analysis, HSFs can be categorized into classes A–C (von Koskull-Döring et al. 2007). Recent studies on *Arabidopsis* have supported that heat shock factor A1 (HsfA1), as a critical regulator, activates heat stress response signaling (Liu and Charng 2013; Ohama et al. 2017; Shi et al. 2015). In *Arabidopsis,* the messenger RNA (mRNA) accumulation levels of heat shock proteins in *HSFA1a*/*b* double mutants and *HSFA1a*/*b*/*d*/*e* quadruple mutants were lower than the corresponding levels in wild-type under heat shock conditions. These results indicated that HsfA1 activates the heat stress transcription network in the early stage of the heat response (Lohmann et al. 2004; Yoshida et al. 2011). The mRNA transcript level of *HSFA2* target genes such as *heat shock proteins 70* and *90* was downregulated in *hsfa2*-knockout mutants under heat stress (Schramm et al. 2006). The *hsfa2*-knockout *Arabidopsis* mutant was sensitive to high temperatures and exhibited decreased hypocotyl growth under prolonged heat stress and recovery (Charng et al. 2007). Overexpression of the transcript of *OsHsfA2e* (LOC_Os03g58160) increased the survival rate of *Arabidopsis* plants after heat stress (Yokotani et al. 2008). Microarray analysis revealed that the expression levels of *OsHsfA2a*, *d*, and *e* were higher in rice seedlings under treatment heat stress than in controls (Chauhan et al. 2011).

Recent research has discovered that ethylene signaling is involved in the heat stress response pathway. *ERF1*-overexpressing *Arabidopsis* exhibited greater tolerance than did controls under heat shock treatment. Microarray analysis revealed that the transcript levels of *HsfA3* and *HSP70* were upregulated in *ERF1*-overexpression line under heat shock treatment. These results supported that ERF1 can recognize the GCC box element on the promoter of *HsfA3* and *HSP70* and can enhance thermotolerance in *Arabidopsis* (Cheng et al. 2013).

Limited information is available on the ethylene signaling regulatory mechanism under heat stress. In the current study, our results showed that the levels of cell membrane oxidation and ion leakage were reduced in rice seedlings under heat treatment combined with ethylene precursor treatment, thereby conferring enhanced thermotolerance. The transcript levels of *HsfA1a* and *A2a, c, d, e, and f* genes were higher under heat treatment combined with ethylene precursor treatment than under heat stress only. Furthermore, the transcript levels of ethylene-signaling-related genes were higher under heat treatment combined with ethylene precursor treatment than under heat stress only. These results supported that ethylene signaling is involved in the complex regulation of *Hsf* gene expression during heat stress.

## Methods

### Plant materials and treatments

*Oryza sativa* L. *japonica* cv. Taikeng No. 9 was used in this study. We used 3% sodium hypochlorite to sterilize the seed surface for 40 min, and the seeds were then washed with sterile deionized water several times to remove the buffer added for seed sterilization. The seeds were placed on filter paper for 3 days and germinated in a growth chamber at 28 °C under 16-h light and at 23 °C under 8-h darkness. The germinated seeds were planted on a metal grid over a 500 mL beaker containing Kimura B solution (Yoshida et al. 1976). A total of 30 germinated seeds were placed in each beaker and the culture solution was changed every 2 days for 12 days. For high-temperature treatment were modified from research in rice (Fang et al. 2015; Mangrauthia et al. 2017), 12-day-old seedlings were placed in a growth chamber at 45 °C under a 16:8-h light:dark cycle, and the relative humidity at 50% for 4 days. For the high temperature combined with ACC treatment, 12-day-old seedlings were treated with 10 μM ACC at 45 °C. Control plants remained in the growth chamber at 28 °C under 16-h light and 23 °C under 8-h darkness. After each treatment, shoot samples were collected from rice seedlings, immediately frozen in liquid nitrogen, and stored at − 80 °C until analysis. Independent experiments with 30 seedlings were conducted and each experiment was repeated at least three times.

### Measurement of fresh weight, survival rate, lipid peroxidation, and ion leakage in rice seedlings

To measure the fresh weight, we collected the shoot of rice seedling and detected the weight per plant after heat stress treatment with or without ACC for 4 days. Independent experiments with 10 seedlings were conducted and each experiment was repeated at least three times. To evaluate the survival rate, growth of new leaves in rice seedlings was allowed after heat treatment with or without 10 μM ACC for 4 days, with recovery for 7 days. The lipid peroxidation assay was conducted according to a modification of the method of Jambunathan (2010). After each treatment, the shoot of rice seedlings was collected 0.2 g fresh weight, homogenized in 4 mL of 5% trichloroacetic acid (TCA), and centrifuged at 10,000×*g* for 5 min. After centrifugation, 1 mL supernatant was collected and mixed with 4 mL thiobarbituric acid (TBA) solution containing 0.5% TCA and 20% TBA. The reaction mixture was heated to 95 °C for 30 min and placed on ice to halt the reaction. Absorbance of the supernatant was detected at 532 and 600 nm on a spectrophotometer (Metertec SP8001, Taipei, Taiwan, R.O.C). The percentage of ion leakage was determined according to the method of Jambunathan (2010) with some modifications. Five leaves of rice seedlings were collected in a 15-mL centrifuge tube with deionized water and incubated at 25 °C for 3 h. After incubation, the conductivity of the bathing solutions of each treatment (C_1_) and deionized water (C_1_′), which was used as the background solution, was determined using an electrical conductivity meter. The bathing solutions of each treatment and deionized water were boiled for 30 min and the conductivity of the bathing solutions of each treatment (C_2_) and deionized water (C_2_′) was determined to calculate the percentage of ion leakage as follows: ion leakage (%) = (C_1_ − C_1_′)/(C_2_ − C_2_′) × 100%.

### Measurement of chlorophyll concentration in rice seedlings

After each treatment, the shoot of rice seedlings was collected and extracted from 50 mg of leaf tissue in 2 mL of 50 mM sodium phosphate buffer (pH 6.8). Then taken 40 μL extraction buffer added into 960 μL of 99% ethanol mix together; the mixture was vortexed thoroughly and incubated for 30 min at 4 °C in darkness. After centrifugation at 4 °C for 15 min at 1000×*g*, the supernatant was collected. Absorbance of the supernatant was measured at 665 and 649 nm on the spectrophotometer (Metertec SP8001, Taipei, Taiwan, R.O.C).

### Assay of H_2_O_2_ accumulation and cell viability by histochemical staining method

Accumulation of H_2_O_2_ in cell was visualized by the 3, 3′-diaminobenzidine (DAB) staining method. 12-day old seedlings after heat stress with or without 10 µM ACC treatment for 4 days, the 3rd leaves were immersed into DAB solution prepared in at RT under dark for 12 h. When brown spots or pattern appeared, we used 75% ethanol and boiled leaves until the pigments were removed from leaves. Cell viability assay was visualized by the Evans blue staining. 12-day old seedlings after heat stress with or without 10 µM ACC treatment for 4 days, and collected the 3rd leaves. To weigh and immerse the leaves into 0.2% Evans blue solution for 12 h. When blue spots or pattern appeared, we used 75% ethanol and boiled leaves until the pigments were removed from leaves. Experiments were repeated three times independently.

### Antioxidant enzyme activity assays

For antioxidant enzyme assays, 12-day-old seedlings were treated with heat stress (45 °C) with or without 10 μM ACC for 4 days and their shoots were collected for analysis. Shoot tissue (150 mg) was homogenized in liquid nitrogen and immediately used for enzyme extraction. The protein concentration of each extract from samples was determined by Bradford protein assay (Bradford 1976). Antioxidant enzyme assay was used 1 mg protein of each extract samples, subsequently. Activity levels of CAT, APX, SOD, and POX were analyzed as previously described (Wu and Yang 2016). In the CAT assay, the decrease in the hydrogen peroxide level was monitored by measuring absorbance at 240 nm for 1 min. Sodium phosphate buffer (50 mM; pH 6.8) was used to extract samples, which were then centrifuged at 12,000×*g* for 20 min. The reaction mixture consisted of 100 mM potassium phosphate buffer (pH 7.0), 1 M H_2_O_2_, and supernatant of the extracted enzyme. H_2_O_2_ was added to initiate the reaction, which was recorded for 1 min. One unit of CAT activity was defined as consumption of 1 nmol H_2_O_2_ per minute. In the APX assay, a decrease in absorbance due to ascorbic acid was recorded at 290 nm (Nakano and Asada 1981). The reaction mixture consisted of 150 mM potassium phosphate buffer (pH 7.0), 1.5 mM ascorbic acid, 0.75 mM ethylenediaminetetraacetic acid (EDTA), 6 mM H_2_O_2_, and supernatant of the extracted enzyme. The decrease in absorbance was measured at 290 nm for 1 min. One unit of APX activity was defined as consumption of 1 μmol ascorbic acid per minute. In the SOD assay, suppression of the oxidation rate of β-nicotinamide adenine dinucleotide (β-NADH) to 50% was monitored by measuring absorbance at 340 nm for 10 min. Sodium phosphate buffer (50 mM; pH 7.4) was used to extract samples, which were then centrifuged at 15,000×*g* for 30 min. The reaction mixture consisted of 100 mM triethanolamine–diethanolamine buffer (pH 7.4), 100 mM/50 mM EDTA/MnCl_2_ (pH 7.4), 7.5 mM β-NADH, 10 mM 2-mercaptoethanol, and supernatant of the extracted enzyme. In the POX assay, production of tetraguaiacol was monitored by measuring absorbance at 470 nm for 1 min. Potassium phosphate buffer (50 mM; pH 5.8) containing 0.8 M KCl was used to extract samples, which were then centrifuged at 12,000×*g* for 20 min. The reaction mixture consisted of 50 mM potassium phosphate buffer (pH 5.8), 21.6 mM guaiacol, 39 mM H_2_O_2_, and supernatant of the extracted enzyme.

### Quantitative RT-PCR analyses

Total RNA was extracted from frozen rice shoots in each treatment by using the TRI reagent (Invitrogen) and the RNA pellet was washed with ice-cold 75% ethanol, air dried, and dissolved in 30 μL diethyl pyrocarbonate water. To prevent DNA contamination, all DNA was removed from the samples by using the TURBO DNA-free Kit (Ambion, Austin, TX, USA). RNA concentration was determined spectrophotometrically and the 260/280-nm absorbance ratio showed expected values between 1.8 and 2.0. Total RNA (2 μg) was reverse transcribed into cDNA by using Moloney murine leukemia virus reverse transcriptase (Invitrogen). Quantitative RT-PCR was performed on a Bio-Rad instrument (CFX Connect™, USA) with SYBR Green dye (Invitrogen) in accordance with the manufacturer’s recommendations. The ubiquitin gene (*Os03g13170*) was used as an internal control to normalize cDNA levels. The amplification conditions were as follows: 94 °C for 5 min, and then 45 cycles at 94 °C for 30 s, 55 °C for 30 s and 72 °C for 30 s. The relative expression levels were analyzed using the 2-ΔΔCt method with Bio-Rad software. Experiments were repeated six times independently and performed in duplicate. The primer sequences used for quantitative RT-PCR analyses are listed in Additional file 1: Table S1.

## Results

### Ethylene-mediated signaling reduced oxidative damage in rice seedlings under heat stress

**Fig. 1:**
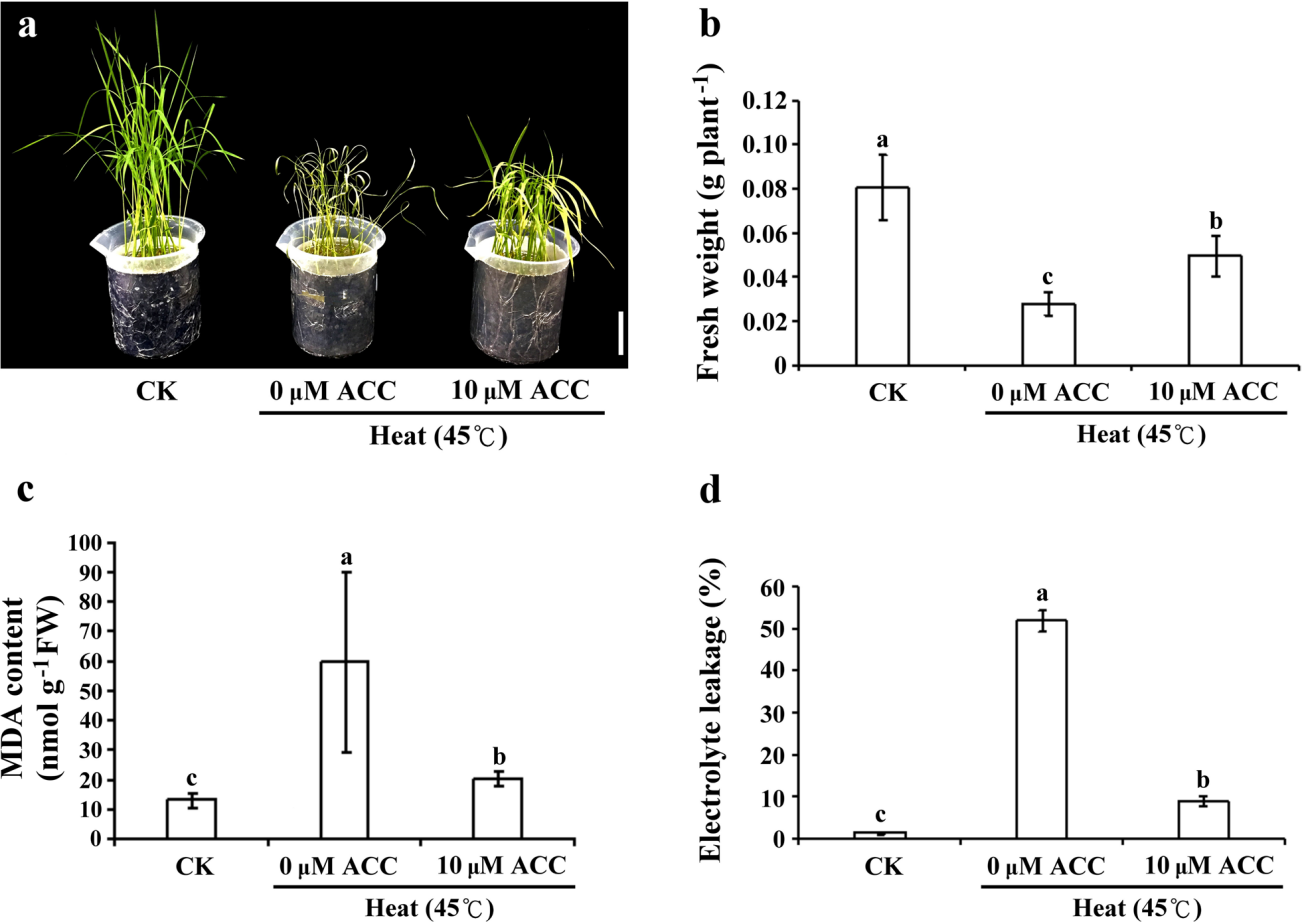
Effects of heat stress with ACC treatment on phenotype and physiological properties of rice seedlings. **a** Phenotypes of 12-day-old rice seedlings exposed to heat treatment at 45 °C with or without 10 μM ACC for 4 days. The same results were observed for three independent experiments. Bar = 5 cm. Fresh weight (**b**), malondialdehyde (MDA) content (**c**), and electrolyte leakage (**d**) in rice seedlings exposed to heat treatment at 45 °C with or without 10 μM ACC for 4 days. Control check (CK). Values are presented as mean ± standard deviation based on 30 seedlings of each treatment obtained from three biologically independent experiments. Different letters indicate significant difference between the treatments assessed using the LSD post hoc test (*P *< 0.05)

To determine the effect of ethylene signaling during heat stress, 12-day-old rice seedlings were grown in Kimura B medium with or without supplementation of ethylene precursor 1-aminocyclopropane-1-carboxylic acid (ACC) and were subsequently subjected to heat stress (45 °C) treatment for 4 days. Regarding their phenotype, rice seedlings under heat stress presented more green leaves after 10 μM ACC treatment (Fig. 1a). Compared with the controls, the fresh weight of rice seedlings showed 63% and 38% reduction after heat stress with 0 and 10 μM ACC treatment for 4 days, respectively (Fig. 1b). Furthermore, we detected the content of malondialdehyde (MDA) as a cell membrane damage indicator. Compared with the control, the MDA content increased 4.5 times under heat stress for 4 days. Compared with the control, the MDA content increased only 1.5 times under heat stress with 10 μM ACC treatment for 4 days (Fig. 1c). Compared with the control, ion leakage was 50% and 7% under heat stress without and with ACC, respectively (Fig. 1d). These results indicated that ethylene-mediated signaling is involved in maintaining water content and significantly reducing intracellular lipid peroxidation in rice seedlings under heat stress.

### Thermotolerance and chlorophyll content were enhanced in rice seedlings under heat stress and ethylene precursor treatment

**Fig. 2:**
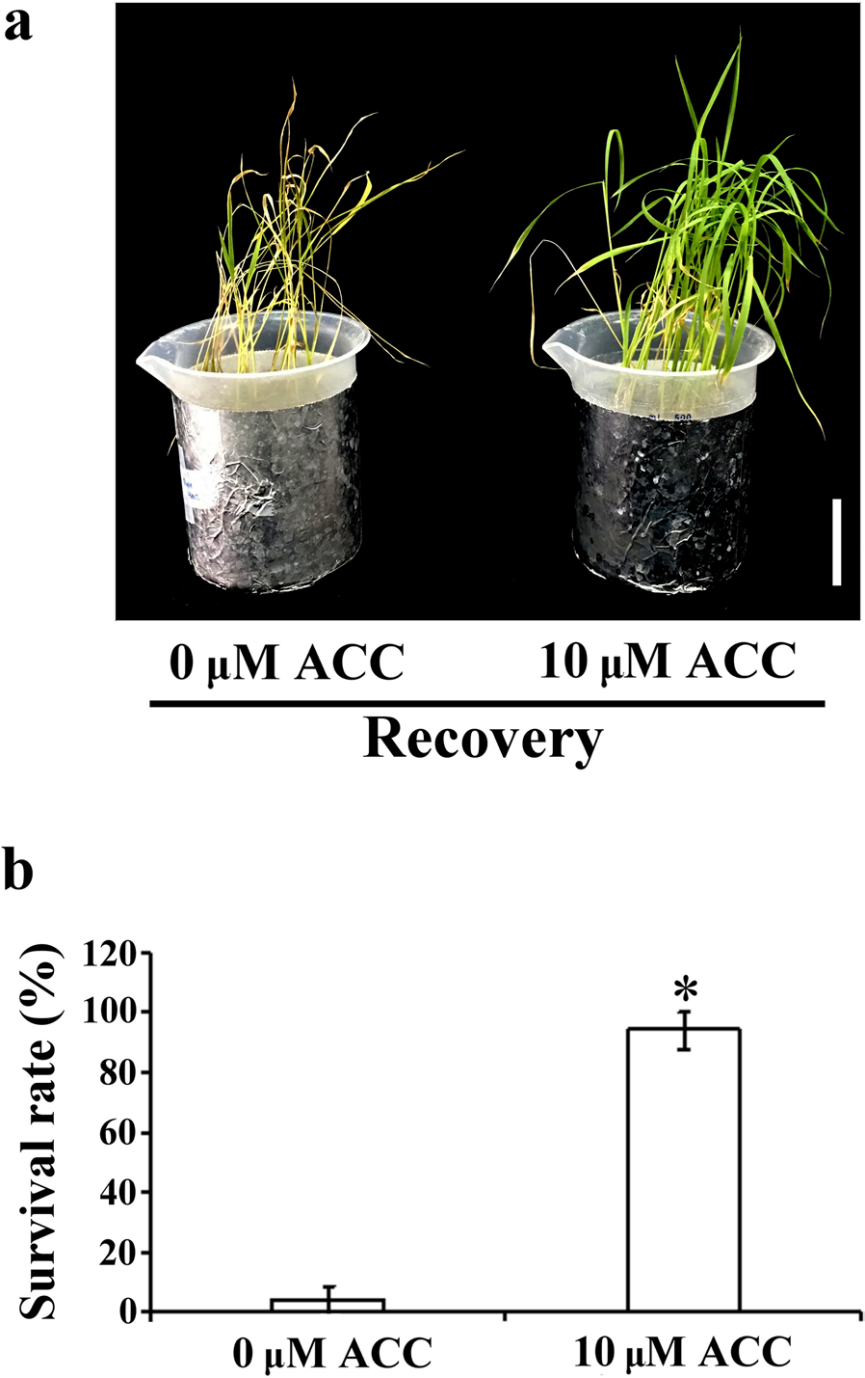
Tolerance determination of rice seedlings under heat stress combined with ACC treatment. **a** Phenotypes of 12-day-old TK9 rice seedlings exposed to 45 °C heat treatment with or without 10 μM ACC for 4 days followed by recovery for 7 days. The same results were observed for three independent experiments. Bar = 5 cm. **b** Survival rate was determined after each treatment and recovery for 7 days. Bar = 5 cm. The values are presented as mean ± standard deviation based on 30 seedlings of each treatment obtained from six biologically independent experiments. *P < 0.05 versus value at 0 μM ACC treatment (Student’s *t* test)

To investigate whether ethylene-mediated signaling affected the seedlings’ tolerance to heat stress, 12-day-old rice seedlings were treated with heat stress with or without ACC for 4 days followed by recovery for 7 days for the survival assay. The survival rates of the rice seedlings were 3.84% and 94.25% when treated with heat stress without or with 10 μM ACC treatment, respectively (Fig. 2).

Chlorophyll is a light-harvesting complex that converts sunlight into chemical energy in a process known as photosynthesis (Bricaud et al. 1995). This study investigated the effect of the ethylene precursor on chlorophyll content in rice seedlings under heat stress with or without 10 μM ACC treatment for 4 days. The results showed that the chlorophyll concentration decreased significantly in rice seedlings under heat stress with 0 μM ACC treatment compared with seedlings under heat stress with 10 μM ACC treatment. No significant difference was observed in chlorophyll b or total content between 0 μM ACC and 10 μM ACC treated seedlings under heat stress (Table 1). Additionally, the chlorophyll a/b ratio decreased significantly in the 0 μM ACC treated rice seedlings compared with the 10 μM ACC treated rice seedlings under heat stress. These results indicated that ethylene-mediated signaling is involved in maintaining chlorophyll content and further enhances thermotolerance in rice seedlings under heat stress.

**Table 1:** Chlorophyll concentration of rice seedlings under heat stress combined with ACC treatment

Concentration (μg g^−1^ FW)	Treatment		
	Control	Heat (45 °C)	
		0 μM ACC	10 μM ACC
Chlorophyll a	0.627 ± 0.081^a,*^	0.354 ± 0.028^c^	0.433 ± 0.010^b^
Chlorophyll b	0.197 ± 0.031^a^	0.198 ± 0.013^a^	0.177 ± 0.004^a^
Total chlorophyll	0.833 ± 0.113^a^	0.558 ± 0.017^b^	0.616 ± 0.006^b^
Chlorophyll a/b ratio	3.196 ± 0.175^a^	1.798 ± 0.268^c^	2.448 ± 0.109^b^

The values are presented as mean ± standard deviation based on six biologically independent experiments

*Different letters indicate significant difference between the treatments assessed using the LSD post hoc test (*P *< 0.05)

### Reduction of the antioxidant response caused by heat stress was ameliorated by ethylene precursor treatment in rice seedlings

**Fig. 3:**
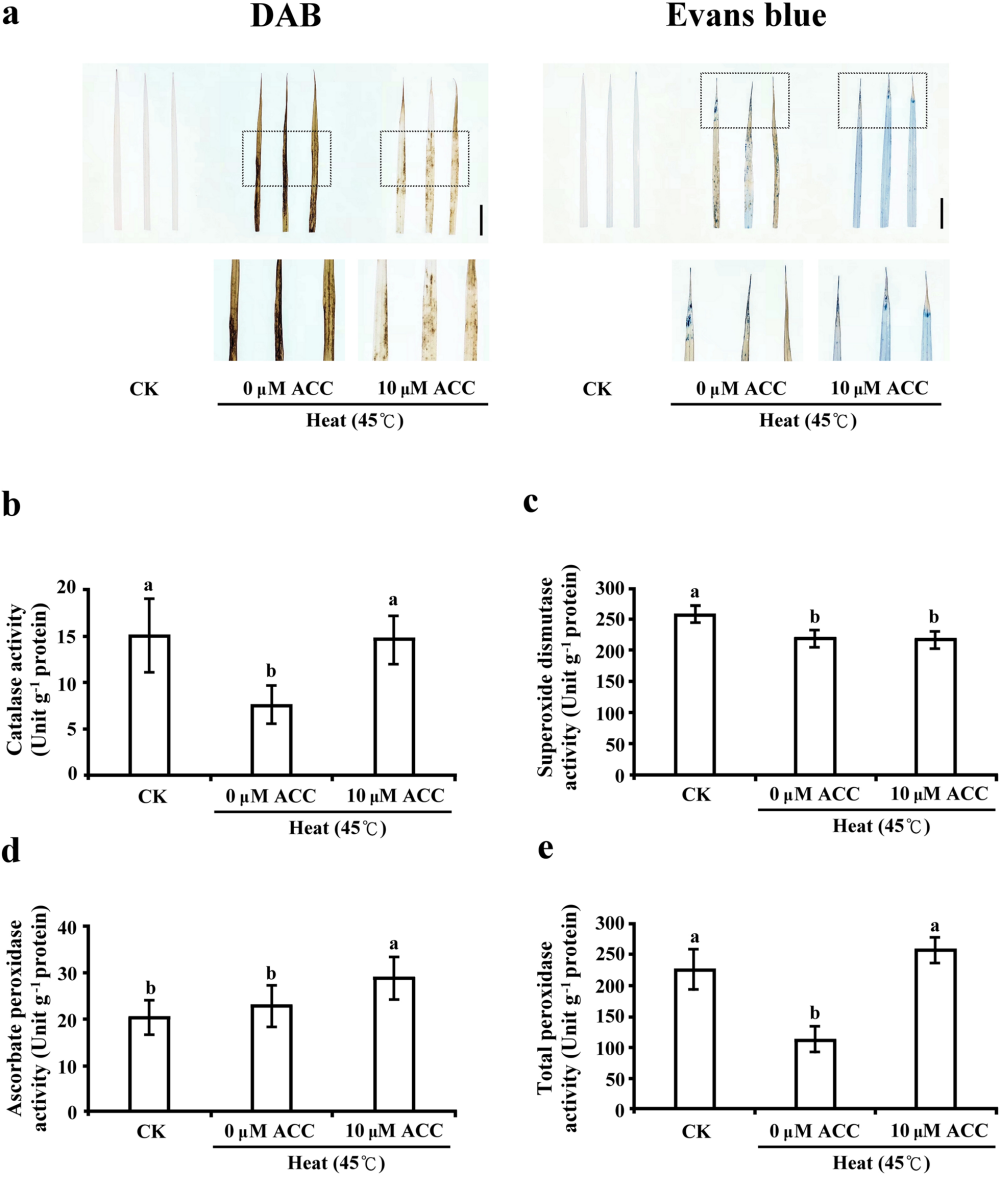
ROS toxicity, cell death and antioxidant enzyme activity assay in rice seedlings under heat stress combined with ACC treatment. **a** DAB staining of H_2_O_2_ and evans blue staining of cell death in detached leaves of 12 days-old rice seedlings after treated 45 °C heat stress with or without 10 µM ACC for 4 days. Bar = 2 cm. Enzyme activity of catalase (**b**), ascorbate peroxidase (**c**), superoxide dismutase (**d**), and total peroxidase (**e**) in the shoots of 12-day-old rice seedlings was detected after 45 °C heat treatment with or without 10 µM ACC for 4 days. Control check (CK). Values are presented as mean ± standard deviation based on 30 seedlings of each treatment obtained from six biologically independent experiments. Different letters indicate significant difference between the treatments assessed using the LSD post hoc test (*P *< 0.05)

Many studies have demonstrated that reactive oxygen species (Choudhury et al. 2017) cause plant cell oxidation under abiotic stress. The cellular scavenging capacity of antioxidant enzymes is important to the metabolism of ROS in sorghum, maize, and wheat plants under heat stress (Djanaguiraman et al. 2010; Sairam et al. 2002; Zhu et al. 2010). We investigate the H_2_O_2_ accumulation by DAB staining and cell viability by evens blue staining in rice seedlings under heat stress with or without ACC treatment. The results of DAB staining showed more H_2_O_2_ accumulation pattern in the leave blades under 0 μM ACC treatment compared with 10 μM ACC treatments under heat stress. In the evans blue staining, the results showed significant accumulation pattern in the leave tip and blade under 0 μM ACC treatment compared with 10 μM ACC treatment (Fig. 3a). Under heat stress, our data displayed less ROS accumulation and cell damage in rice seedling by ethylene signaling. In the present study, to evaluate the effect of ethylene precursor on antioxidant enzyme activity under heat stress, we determined the activity of catalase (CAT), superoxide dismutase (SOD), ascorbate peroxidase (APX), and total peroxidase (POX) in rice seedlings under heat stress with or without ACC treatment for 4 days. Our results showed that reductions in CAT and POX activity caused by heat stress were ameliorated by 10 μM ACC treatment (Fig. 3b, e). Activity of APX was higher in the 10 μM ACC treated rice seedlings than in the 0 μM ACC treated and control seedlings (Fig. 3d). However, the activity of SOD presented no significant difference under heat stress with or without ACC treatment, but lower than control seedlings (Fig. 3c). Our data showed that the ethylene precursor affected antioxidant enzyme activity under heat stress.

### Ethylene-mediated signaling enhanced *Hsf* and ethylene-related gene expression in rice seedlings under heat stress

**Fig. 4:**
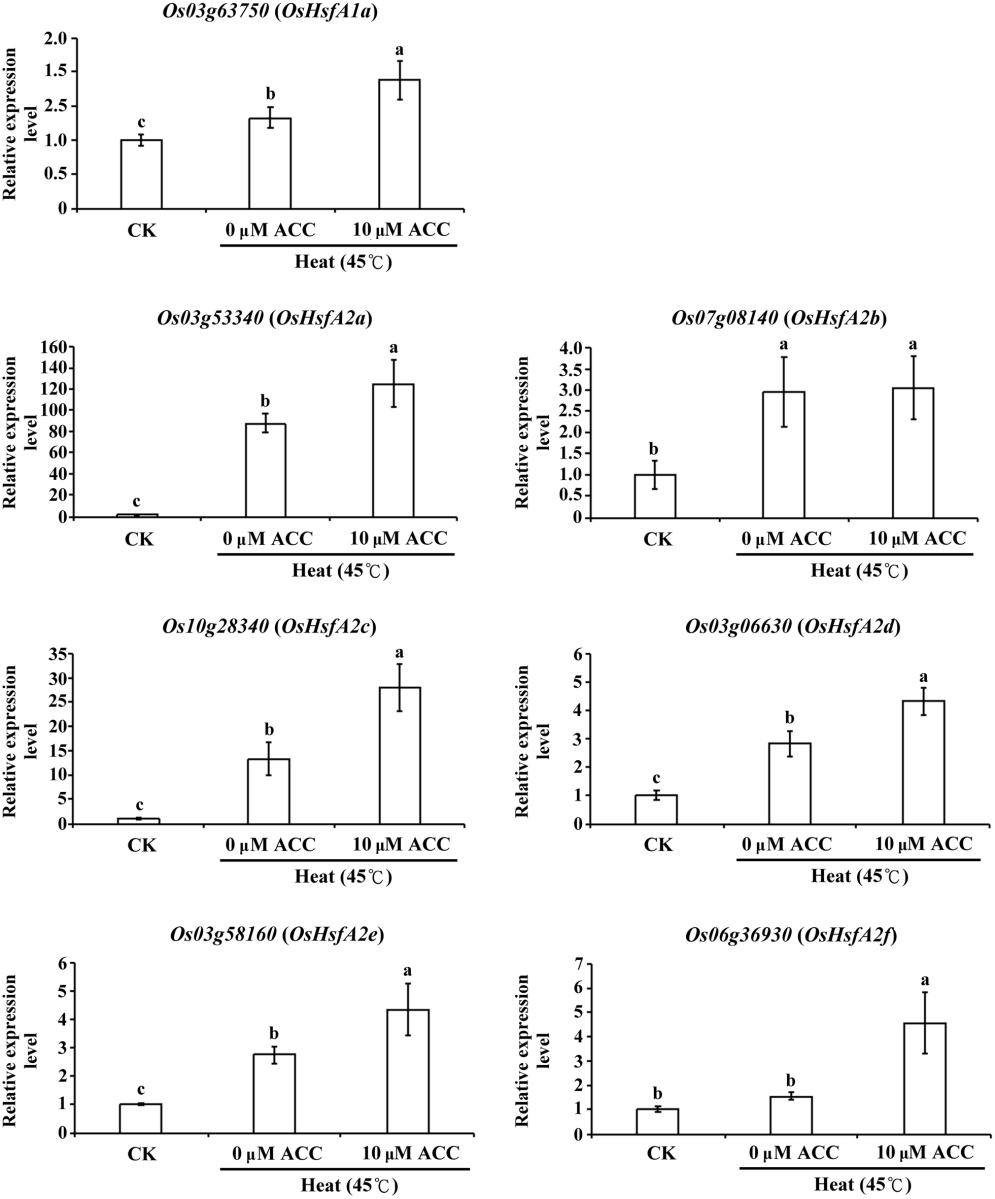
Transcript levels of heat shock factor genes under heat stress with ACC treatment. Total RNA was isolated from the shoots of 12-day-old seedlings after heat stress (45 °C) with or without 10 µM ACC for 1 h. Relative expression levels of *Os03g63750* (*HsfA1a*), *Os03g53340* (*HsfA2a*), *Os07g08140* (*HsfA2b*), *Os10g28340* (*HsfA2c*), *Os03g06630* (*HsfA2d*), *Os03g58160* (*HsfA2e*), and *Os06g36930* (*HsfA2f*) were determined through quantitative RT-PCR. Control check (CK). Values are presented as mean ± standard deviation based on 30 seedlings of each treatment obtained from six biologically independent experiments. Different letters indicate significant difference between the treatments assessed using the LSD post hoc test (*P *< 0.05)

To clarify the effects of ethylene signaling on *Hsf* expression during heat stress, 12-day-old rice seedlings were treated with heat stress with or without 10 µM ACC treatment for 1 h and then subjected to quantitative reverse transcriptase-polymerase chain reaction (RT-PCR) to determine mRNA expression. The results indicated that in addition to *HsfA2b*, the induction levels of *HsfA1a*, *HsfA2a*, *c*, *d*, *e*, and *f* were significantly higher in seedlings under heat stress with 10 μM ACC treatment than in those under heat stress only (Fig. 4).

**Fig. 5:**
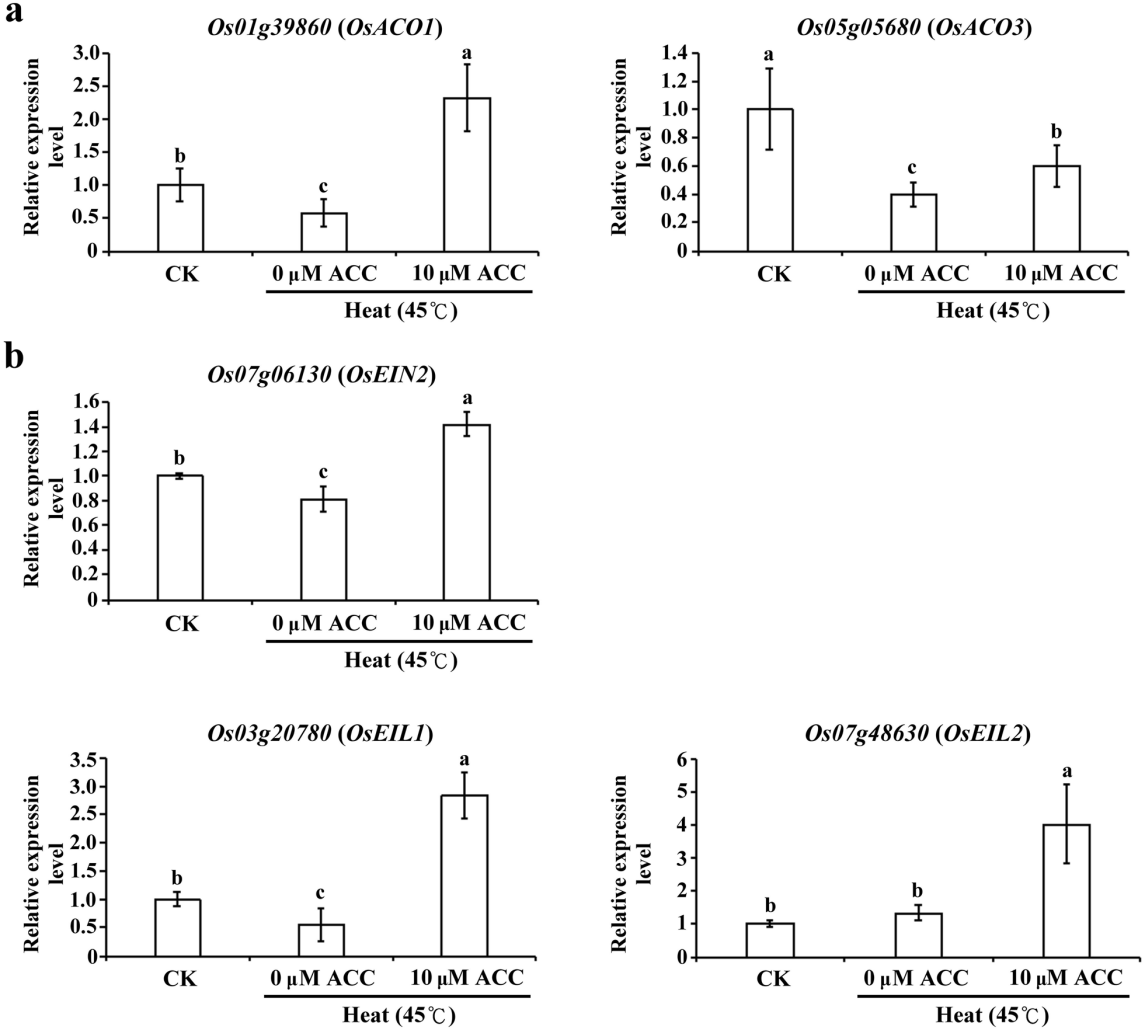
Transcript levels of ethylene biosynthesis related genes and ethylene signaling related genes under heat stress with ACC treatment. Total RNA was isolated from the shoots of 12-day-old seedlings after heat stress (45 °C) with or without 10 µM ACC for 1 h. Relative transcript levels were analyzed using quantitative RT-PCR. **a** Relative gene expression levels of *Os01g39860* (*ACC oxidase 1* [*ACO1*]) and *Os05g05680* (*ACC oxidase 3* [*ACO3*]) genes associated with ethylene biosynthesis pathways were determined using quantitative RT-PCR. **b** Relative gene expression levels of *Os07g06130* (*ethylene insensitive 2* [*EIN2*]), *Os03g20780* (*ethylene insensitive*-*like 1* [*EIL1*]), and *Os07g48630* (*ethylene insensitive*-*like 2* [*EIL2*]) genes associated with ethylene signaling pathways were determined using quantitative RT-PCR. Control check (CK). The data represent average values ± standard deviations based on 30 seedlings of each treatment obtained from six biologically independent experiments. Different letters indicate significant difference between the treatments assessed using the LSD post hoc test (*P *< 0.05)

In the ethylene biosynthesis process, ACC oxidase, as an impotent enzyme, was catalyzed ACC into ethylene. The quantitative RT-PCR results showed the mRNA expression of *ACC oxidase 1* (*ACO1*) and *ACC oxidase 3* (*ACO3*) were significantly higher in seedlings under heat stress with 10 µM ACC treatment than under heat stress only (Fig. 5a). The expression levels of *EIN 2*, *EIN*-*like 1*, and *EIN*-*like 2* (*OsEIN2*, *OsEIL1*, and *OsEIL2*, respectively, involved in ethylene signaling) were significantly higher in seedlings under heat stress with 10 μM ACC treatment than in those under heat stress only (Fig. 5b). These results indicated that ethylene-mediated signaling affected expression of *Hsfs* and genes involved in ethylene biosynthesis and signaling in rice seedlings under heat stress.

## Discussion

In plants, heat stress causes water deficit, cell dehydration, and reduced leaf expansion. The optimal temperature for rice growth is 28/22 °C (Prasad et al. 2006). During reproductive processes, high temperature causes the failure of anther dehiscence, pollen grain germination on stigma, and pollen tube elongation, all of which lead to rice plant sterility and reduce rice yield (Satake and Yoshida 1978). Ethylene, a stress hormone, is involved in the regulation of many physiological properties. Studies have reported that ethylene is involved in the regulation of basal thermotolerance in *Arabidopsis* under heat stress (Larkindale and Knight 2002; Larkindale et al. 2005). Under normal condition, 12-day-old rice seedling did not show any difference phenotype between 0 and 10 µM ACC treatment after treated for 4 days. In the present study, rice seedlings under heat stress with ACC treatment exhibited less yellowish and withered phenotype (Fig. 1a, b). The lipid oxidation of the cell membrane caused by heat stress was accompanied by electrolyte leakage (Liu and Huang 2000). However, in the present study, cell membrane stability was higher in rice seedlings with ethylene precursor treatment than in those under heat stress only. Studies have revealed lower oxidative damage and higher chlorophyll concentration in creeping bentgrass (*Agrostis stolonifera* var. *palustris*) upon pretreatment with the ethylene precursor ACC and subsequent exposure to heat stress (Larkindale and Huang 2004). In the present study, our results showed that the MDA concentration and electrolyte leakage were reduced by ethylene-mediated signaling in rice seedlings under heat stress with ACC treatment (Fig. 1c, d). Furthermore, we showed that heat stress combined with ACC treatment induced ethylene-mediated signaling, which resulted in higher survival rates in rice seedlings with the combination treatment than in seedlings under heat stress only (Fig. 2).

Chlorophyll a is the primary photosynthetic pigment that absorbs blue, red, and violet wavelengths in the visible spectrum. The primary function of chlorophyll b is to complement the absorption spectrum of chlorophyll a by extending the range of light wavelengths (Bricaud et al. 1995). In a high-temperature environment, injury of the thylakoid membrane has been demonstrated to reduce chlorophyll content in rice and maize (Ristic et al. 2007). The chlorophyll a/b ratio is associated with the photosynthesis capability of plants under biotic or abiotic stress. In this study, our results showed that ethylene-mediated signaling was involved in maintaining chlorophyll a content; thus, rice seedlings under heat stress and ACC treatment showed a higher chlorophyll a/b ratio than did those under heat stress only (Table 1).

ROS such as hydrogen peroxide, hydroxyl radical, superoxide anion radicals, and singlet oxygen can be generated and accumulated in plants under heat stress (Suzuki et al. 2012). Uncontrolled ROS accumulation can damage a cell membrane, protein, and DNA and then cause programed cell death. The detoxification enzyme system is involved in the tolerance of plants under biotic and abiotic stress (Choudhury et al. 2017; Larkindale and Huang 2004). The results of the present study demonstrated that the enzyme activity of CAT, APX, and POX was enhanced by ACC treatment under heat stress (Fig. 3), and the findings confirmed that the ethylene signaling pathway can regulate scavenger enzymes to enable rice seedling adaptation under oxidative stress caused by high temperatures.

HsfA1s and HSFA2s play key roles in activating the heat stress transcription network (Ohama et al. 2017). Although studies have reported that the transcript levels of rice *HSFA2a*, *d*, and *e* are upregulated under heat stress, little is known about the involvement of ethylene-mediated signaling in the regulation of *Hsf* expression during heat stress (Cheng et al. 2013). In the current study, our results showed that the expression levels of *HSFA1a* and *HSFA2a*, *c*, *d*, *e*, and *f* were higher in rice seedlings under heat stress with ACC treatment than in those under heat stress only (Fig. 4). The genes expression was triggered at the related early stage compare with the phenotype change during stress. The study was shown that expression level of *OsACO1* was no significant different in 14-day-old rice seedling under heat shock 40 °C for 90 min compared with control (Wilkins et al. 2016). In this study, our results showed the gene expression levels of *OsACO1* and *OsACO3* in 12-day-old rice seedlings under 45 °C for 1 h without ACC treatment was lower than control (Fig. 5). However, the expression of *OsHsf A1* and *OsHsf A2* genes were enhanced under 45 °C for 1 h without ACC treatment condition (Fig. 4). The results implied that the expression of *OsHsfA1* and *OsHsfA2* genes may regulated by complex signaling pathways during heat stress. One study depicted a model of the ethylene downstream signaling pathway, which indicated that the ethylene-mediated signaling was involved in maintaining the stay-green phenotype in *Arabidopsis* under heat stress (Abdelrahman et al. 2017). In the present study, we showed that expression of *OsEIN2*, *OsEIL1,* and *OsEIL2* associated with ethylene signaling pathways was increased under heat stress with ACC treatment (Fig. 5). These data imply that ethylene-mediated signaling is involved in the expression of *HSFs* and downstream ethylene-signaling-related genes, thereby increasing the thermotolerance of rice seedlings.

Under heat stress, our study indicated the transcript level of ethylene biosynthesis gene and signal transduction associated gene were up-regulated and expanded the ethylene signal response via applied ethylene precursor under heat stress. The expression of *HSFs* gene was also up-regulated by ethylene-mediated signaling. We speculated the Hsfs triggering the antioxidant mechanism and reducing the oxidative damage during heat stress. Furthermore, the thermotolerance was enhanced and presented higher survival rate via ethylene-mediated signaling in rice seedling after heat stress.

## Conclusions

In this study, our results suggest that ethylene-mediated signaling is involved in the maintenance of chlorophyll content, reduction of oxidative damage, and enhancement of thermotolerance in rice seedlings under heat stress. In addition, ethylene-mediated signaling regulates the mRNA transcripts of *Hsfs* and ethylene-signaling-related genes during heat stress. This study provides key insights that clarify the interactions between ethylene and Hsfs that confer thermotolerance to rice seedlings.

## Supplementary information

**Additional file 1: Table S1.** The primer sequence used for quantitative RT-PCR in this study.

## Data Availability

All data supporting the conclusions of this article are provided within the article (and its additional files).

## Abbreviations

ACC: 1-aminocyclopropane-1-carboxylic acid
APX: ascorbate peroxidase
CAT: catalase
HSF: heat shock factor
MDA: malondialdehyde
POX: total peroxidase
ROS: reactive oxygen species
RT-PCR: reverse transcriptase-polymerase chain reaction
SOD: superoxide dismutase
