# Supplementary material for: Ethylene-mediated signaling confers thermotolerance and regulates transcript levels of heat shock factors in rice seedlings under heat stress
Source: Bot Stud. 2019 Sep 23;60:23. doi: 10.1186/s40529-019-0272-z (PMC6757084; doi:10.1186/s40529-019-0272-z)
Supplement: Supplementary file 1 — Additional file 1: Table S1. The primer sequence used for quantitative RT-PCR in this study. [file 40529_2019_272_MOESM1_ESM.docx]

Table S1

The primer sequence used for quantitative RT-PCR in this study

| Locus number | Forward primer (5'→3') | Reverse primer (5'→3') | Location |
| --- | --- | --- | --- |
| *LOC_Os03g63750* (*HsfA1a*) | 5'-gacaacggcatacccaaaga-3' | 5'-ccacagccttcggaacaga-3' | Exon |
| *LOC_Os03g53340* (*HsfA2a*) | 5'-gcgtccaggagagtaacagc-3' | 5'-gtcatcctcctcgtcgttgt-3' | Exon |
| *LOC_Os07g08140* (*HsfA2b*) | 5'-gggcttgttggtgaggagaac-3' | 5'-catcttctccgacaacacattca-3' | Exon |
| *LOC_Os10g28340* (*HsfA2c*) | 5'-caatgcacctccgtcacaac-3' | 5'-cctcgaatccaaactcaccaa-3' | Exon |
| *LOC_Os03g06630* (*HsfA2d*) | 5'-ctaaggcacaagcagcaaagc-3' | 5'-cgcatgttgcagcctctct-3' | Exon |
| *LOC_Os03g58160* (*HsfA2e*) | 5'-ggcaaggcgcaacaagat-3' | 5'-tctccgttagatggcttgattttc-3' | Exon |
| *LOC_Os06g36930* (*HsfA2f*) | 5'-agacgttcgagatggtggag-3' | 5'-acaccacgaagctgttcctc-3' | Exon |
| *LOC_Os01g39860* (*ACO1*) | 5'-ggactactaccagggcacca-3' | 5'-gctgaagagcttcttgacgg-3' | Exon |
| *LOC_Os05g05680* (*ACO3*) | 5'-ttcgtacgacatgtagtttccg-3' | 5'-ttgttttgtccaaccagcaa-3' | 3’UTR |
| *LOC_Os07g06130* (*EIN2*) | 5'-tagggggactttgaccattg-3' | 5'-tggaagggaccagaagtgtt-3' | 3’UTR |
| *LOC_Os03g20780* (*EIL1*) | 5'-gcaaccagaaggctgttgtt-3' | 5'-ggcatgaggaacttgtcgtt-3' | Exon |
| *LOC_Os07g48630* (*EIL2*) | 5'-gaccatgtacgacaacgacg-3' | 5'-gaaattagcctgctgctgct-3' | Exon |
| *LOC_Os03g13170* (*Ubi*) | 5'-aaccagctgaggcccaaga-3' | 5'-acgattgatttaaccagtccatga-3' | 3’UTR |
